# Developmental dysplasia of the hip is common in patients undergoing total hip arthroplasty under 50 years of age

**DOI:** 10.1051/sicotj/2023020

**Published:** 2023-08-09

**Authors:** Varun Muddaluru, Oliver Boughton, Thomas Donnelly, John O’Byrne, James Cashman, Connor Green

**Affiliations:** 1 National Orthopaedic Hospital Cappagh Finglas Dublin; 2 Children’s Health Ireland at Temple Street Rotunda Dublin

**Keywords:** DDH, Hip dysplasia, Early onset osteoarthritis, Hip arthroplasty, Tönnis angle, Lateral centre-edge angle

## Abstract

*Introduction*: Developmental dysplasia of the hip (DDH) refers to congenital and/or developmental hip instability that can result in hip joint subluxation or dislocation. When detected neonatally, conservative treatment with hip bracing can restore normal hip anatomy. Missed detection of DDH in the neonatal period or late development of DDH often requires surgical intervention to correct the abnormal anatomy. Furthermore, despite surgical intervention, residual sequelae may persist leading to early osteoarthritis of the hip joint requiring joint replacement surgery. *Aim*: This study investigates the prevalence of hip dysplasia in patients undergoing total hip arthroplasty (THA) under 50 years of age. *Methods*: The hip arthroplasty database at a national referral centre was investigated from January 2014 to December 2020. In patients under 50 years of age, those with an adequate pre-operative anteroposterior pelvic radiograph without previous hip arthroplasty were included, while those with inadequate radiographs were excluded. The following measurements were made on the contralateral non-operated hip: (1) lateral centre-edge angle (LCEA), (2) Tönnis angle, (3) acetabular version, (4) acetabular depth, (5) femoral head lateralisation, (6) femoral head extrusion index, and (7) acetabular depth-to-width ratio. *Results*: In total, 451 patients were included in this study. Twenty two percent of the patients had hip dysplasia, based on a LCEA of <25° and 42.6% of patients had hip dysplasia, based on a Tönnis angle of > 10°. The mean LCEA and Tönnis angle were 31.47 ± 9.64 and 9.82 ± 6.79°, respectively. *Conclusion*: Hip dysplasia is common in patients undergoing THA under the age of 50 years with over 40% having dysplasia according to the Tönnis angle. Classification of primary and secondary osteoarthritis in the joint registries will benefit our knowledge on the prevalence of DDH in the adult population.

## Introduction

Developmental dysplasia of the hip (DDH) refers to a broad spectrum of congenital and/or developmental hip disorders including (1) neonatal instability, (2) acetabular or femoral dysplasia, (3) hip subluxation, and (4) frank hip dislocation [1]. The incidence of DDH varies significantly globally with the lowest incidence of 0.06 per 1000 in Africans from Africa to the highest incidence of 76.1 per 1000 in Native Americans [2]. The risk factors include breech presentation, family history, female sex, and swaddling [1]. Screening for DDH usually comprises of the following: (1) universal newborn clinical screening (Barlow and Ortolani manoeuvres) with risk factor assessment and (2) universal ultrasound screening or selective ultrasound screening after positive findings on clinical exam or risk factor assessment [3]. Next, treatment aims to achieve a concentric reduction of the femoral head into the acetabulum, as this stimulates normal acetabular development [1]. When DDH is diagnosed early (below 6 months of age), a concentric reduction can be achieved conservatively, commonly with a Pavlik harness. Failure of the Pavlik harness or late diagnosis (6–8 months or later) requires surgical intervention including closed or open reduction of the dysplastic hip joint, and if need be, acetabular and femoral osteotomy procedures [1].

Despite treatment, residual sequelae of DDH persist in a portion of the patients. Residual dysplasia is present in up to 19% of patients treated successfully with a Pavlik harness and 22–33% of patients treated with a closed or open reduction [4–6]. Residual dysplasia, a missed diagnosis, or late presentation of DDH increases the risk of the development of early secondary hip osteoarthritis in young adulthood [7]. Although the causal mechanism between hip dysplasia and early osteoarthritis has not been established, leading theories suggest abnormal biomechanics and shear forces [8]. Approximately 25–50% of patients with hip dysplasia develop radiographic osteoarthritis by 50 years of age [8]. In comparison with a normal hip, if hip instability is present at birth, the relative risk of total hip arthroplasty (THA) is 2.6. In the US, the estimated prevalence of hip dysplasia is 0.1% in the adult population [7]. In Denmark, DDH represents 2.6–9.1% of total cases of THA and it is the main cause of THA in young adults, representing 21–29% of cases [9, 10].

The prevalence of hip dysplasia in the adult population has not yet been identified in the authors’ country. In this study, we aim to determine the prevalence of adult hip dysplasia in patients undergoing THA under 50 years of age by analysing radiographic parameters.

## Methods

The hip arthroplasty database at a national referral centre was investigated from January 2014 to December 2020. Research ethics committee approval was granted for this study. Patients were included if they were under 50 years of age and had an adequate anteroposterior pelvic radiograph before the hip replacement surgery. Patients were excluded if they had previous hip replacement surgery or if the radiographs were not adequate. The following radiological parameters are of interest when making a radiological diagnosis of hip dysplasia in adults and were made on the contralateral (non-operated on) hip: (1) lateral centre-edge angle (LCEA) of Wiberg (<25° = DDH, 25°–40° = normal, >40° = femoroacetabular impingement), (2) Tönnis angle (<0° = impingement, 0°–10° = normal, >10° = DDH), (3) acetabular version (anteversion = normal; retroversion = associated with hip dysplasia), (4) acetabular depth (coxa profunda or protrusion = impingement), (6) femoral head lateralization (>10 mm = dysplasia), (6) femoral head extrusion index (>0.25 = dysplasia), (7) acetabular depth-to-width ratio (<0.38 = dysplasia) [11, 12]. The methods to measure these radiological parameters were adopted from a paper by Clohisy [13] which describes the radiographic evaluation of the young adult hip. One member of the research team analyzed the radiographs to prevent any inconsistencies and discrepancies caused by multiple evaluators. Hip dysplasia was determined with the evaluation of the LCEA and the Tönnis angle. A LCEA of <25° indicates dysplasia, while a Tönnis angle of >10° indicates dysplasia [13]. In total, 632 radiographs were considered for analysis. One hundred and eighty one patients were removed due to poor radiographs or previous hip arthroplasty, and therefore, radiographs of 451 patients were analyzed for the above-mentioned parameters. Radiographs were accessed and analyzed on the McKesson Radiology platform and data was inputted into Microsoft Excel. Subsequently, data analysis and visualization were performed with RStudio v1.4.1106.

## Results

**Figure 1:**
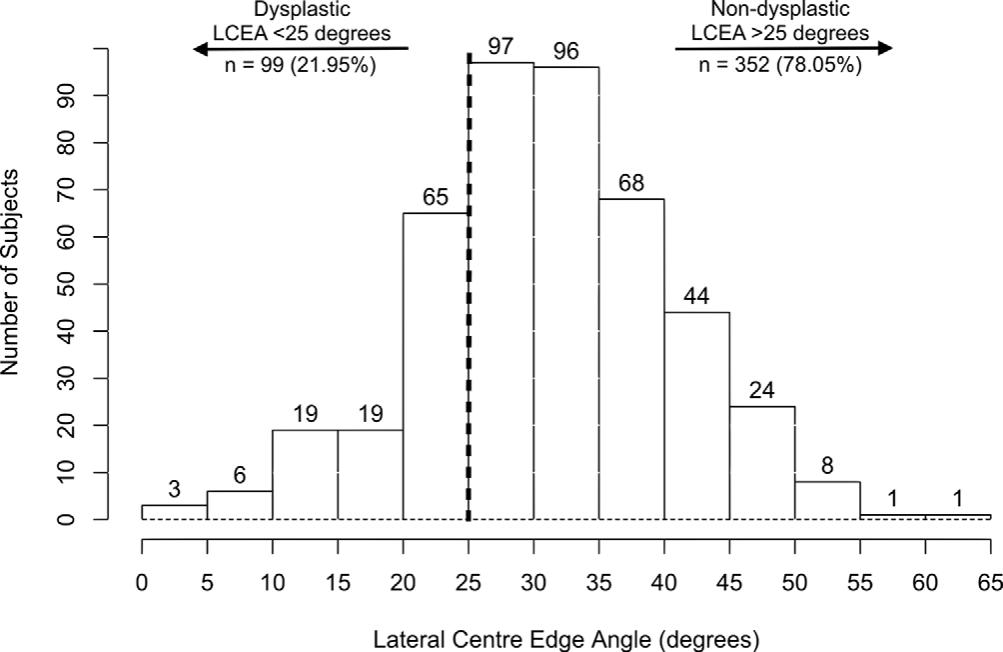
Histogram showing the distribution of subjects according to the lateral centre-edge angle (LCEA). A LCEA of <25° indicates hip dysplasia, while >25° is non-dysplastic.

**Figure 2:**
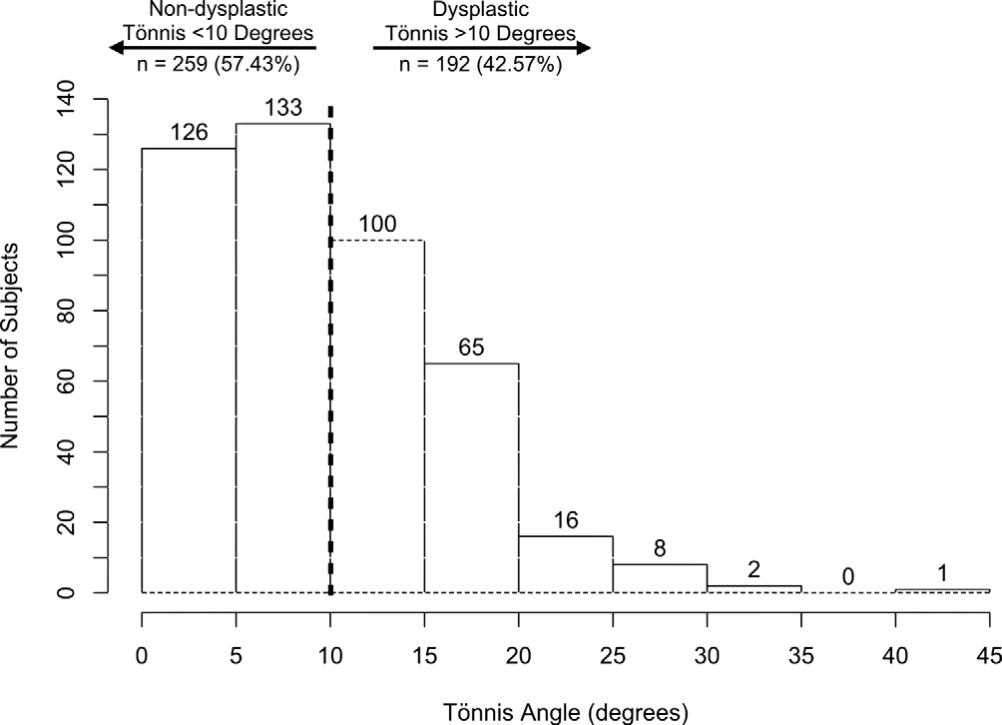
Histogram showing the distribution of subjects according to the Tönnis angle. A Tönnis angle of >10° indicates hip dysplasia, while <10° is non-dysplastic.

In total, anteroposterior pelvic radiographs from 451 patients were analyzed. The mean LCEA was 31.47 ± 9.64° and the mean Tönnis angle was 9.82 ± 6.79° respectively (Table 1). Based on the LCEA, 99 (22%) patients were dysplastic (LCEA < 25°) and 352 (78%) patients were non-dysplastic (LCEA > 25°) (Table 2, Figure 1). Based on the Tönnis angle, 192 (42.6%) patients were dysplastic (Tönnis > 10°) and 259 (57.5%) patients were non-dysplastic (Tönnis < 10°) (Table 2, Figure 2). The mean femoral head lateralization was 11.47 ± 4.85 mm, the mean femoral head extrusion index was 19.94 ± 9.09 and the mean acetabular depth-to-width ratio was 27.46 ± 4.60. For the acetabular version, 361 (80%) patients were anteverted and 90 (20%) patients were retroverted. With regards to acetabular depth, 267 (59.2%) patients were categorized as profunda, 183 (40.6%) as normal, and 1 (0.2%) patient as protrusio.

**Table 1:** Overview of the results of radiological parameters evaluated for hip dysplasia.

	Mean ± *SD*	Median	Range
Lateral centre-edge angle (°)	31.47 ± 9.64	31.00	1.00–62.00
Tönnis angle (°)	9.82 ± 6.79	8.70	0.10–44.00
Femoral head lateralization (mm)	11.27 ± 4.85	11.00	0–27.70
Femoral head extrusion index	19.94 ± 9.09	18.75	0–50.00
Acetabular depth-to-width ratio	27.46 ± 4.60	27.54	3.21–39.71

**Table 2:** Proportion of dysplastic and non-dysplastic subjects according to the lateral centre-edge angle (DDH = <25°) and Tönnis angle (DDH = >10°).

	Dysplastic	Non-dysplastic
	*n* (%)	*n* (%)
Lateral centre-edge angle (DDH = <25°)	99 (21.95)	352 (78.05)
Tönnis angle (DDH = >10°)	192 (42.57)	259 (57.43)

## Discussion

In patients under the age of 50 years undergoing hip replacement surgery, radiographic hip dysplasia is more common than previously thought, with over 40% of the young hip replacement patients having radiographic hip dysplasia, when classified according to the Tönnis angle >10° (Figure 2). The Tönnis angle was chosen as the preferred measurement to determine the presence of radiographic hip dysplasia because, in a previous study comparing 2-dimensional radiographic measurements to 3-dimensional measurements of femoral head coverage using computer tomography, the Tönnis angle was shown to be a better predictor of 3-dimensional femoral head coverage than the LCEA [14]. The prevalence of hip dysplasia in the current study population is higher than in other epidemiological studies. In a cross-sectional survey of 2232 women and 1336 men (age range 20–91 years) conducted by Jacobsen and Sonne-Holm (2005) in Copenhagen, Denmark, the prevalence of hip dysplasia ranged from 5.4% to 12.8% based on the radiographic measurement applied [15]. In another cross-sectional study from Copenhagen by Gosvig et al. (2010), the prevalence of acetabular dysplasia was 4.3% and 3.6% for men and women, respectively, in a study population of 3620 subjects [16]. Next, in a study by Birrell et al. (2003) from the UK, 32% of the 195 subjects (63 male and 132 female, aged 40 or over) who presented to the GP office with hip pain had acetabular dysplasia [17].

**Figure 3:**
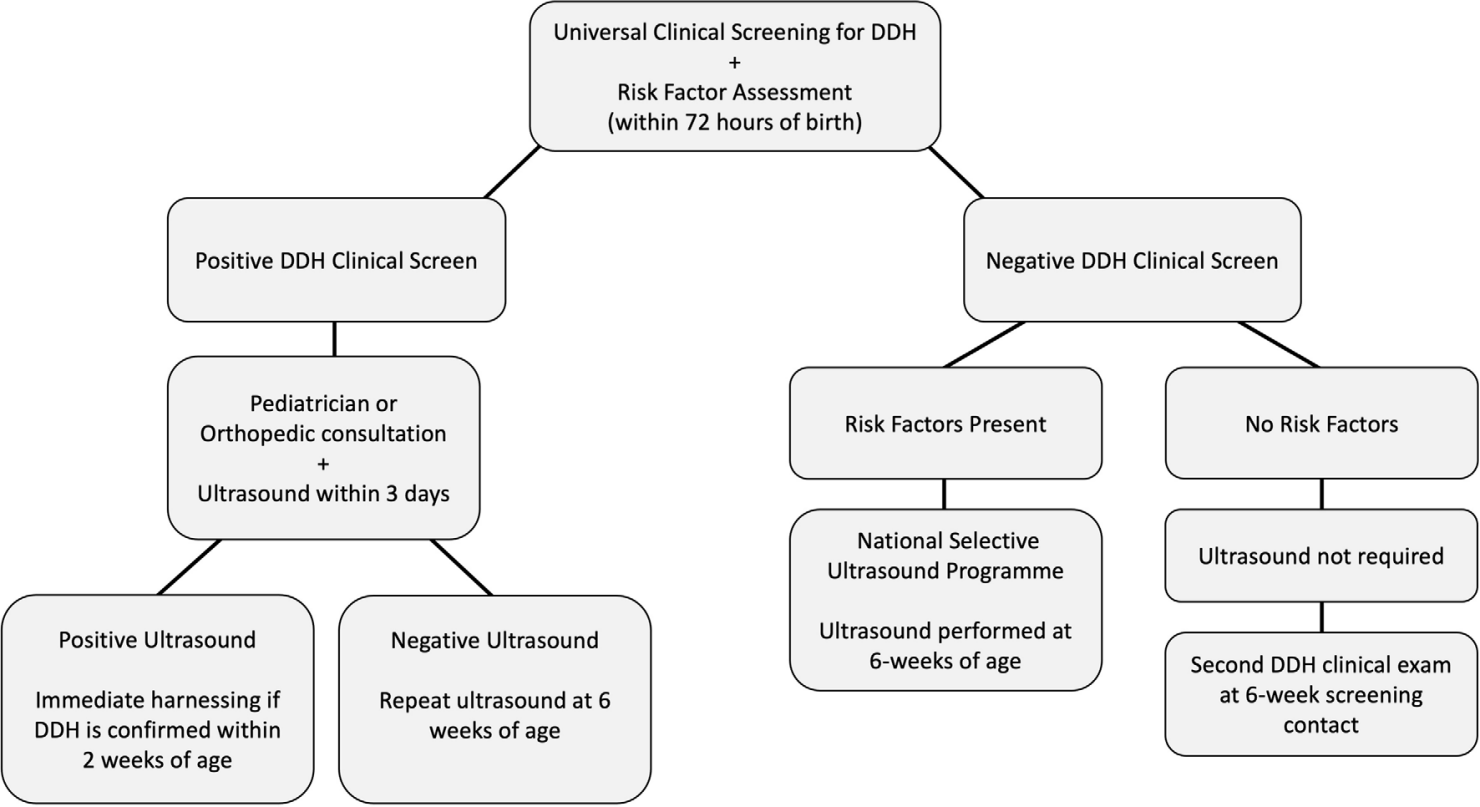
Summary of the newborn clinical screening program for developmental dysplasia of the hip (DDH) in Ireland.

Late diagnosis of DDH or late presenting DDH in adolescence or adulthood requires complex surgical intervention demanding greater resources from the healthcare system for management [18]. In a study evaluating the prevalence of DDH in newborns in the southeast region of Ireland, the annual incidence of late diagnosis of DDH (>3 months of age) was found to be 7.9 per 1000 live births. The mean age of diagnosis was 33.2 weeks (7.6 months), with 79.8% being female and 20.2%, being male new-borns [19]. Additionally, 61 (70.9%) cases of late-diagnosed DDH were identified in the community highlighting the importance of medical staff in the community (primarily Public Health Nurses and General Practitioners) in detecting DDH as well as suggesting improvements to the national screening apparatus to prevent the late diagnosis of DDH [19]. Figure 3 summarizes the clinical screening program for DDH in Ireland. Early diagnosis of DDH (<3 months of age) can be successfully managed non-operatively with a Pavlik harness or abduction braces, while the late diagnosis of DDH (>3 months of age) increases the need for surgical intervention such as a closed or open reduction or a hip preservation surgery including pelvic, acetabular or femoral osteotomy [1]. Woodacre [18] compared the healthcare costs (according to costs in 2008) for the treatment of early and late presentation or diagnosis of DDH in the UK including the screening (clinical and ultrasound) and administrative costs. The treatment for early presentation and diagnosis (<3 months of age) with the Pavlik harness cost £601/child, while late presentation or diagnosis (>3 months of age) treated with surgical intervention costed £4352/child, which is 7 times the cost of treatment with Pavlik harnessing [18]. Furthermore, in early presenting children, in whom the Pavlik harness failed to correct DDH, the subsequent surgical interventions cost £7052/child, 11.7 times greater than treatment with Pavlik harnessing and 1.6 times greater than surgical treatment following the late presentation. The following are unit costs (2008 UK prices) for various treatment modalities: Pavlik harnessing – £35, arthrogram – £151.83, open reduction – £747.91, pelvic osteotomy – £853.93, and femoral osteotomy – £1149.28 [18]. Additional costs include pre-assessment visits, inpatient hospital stays, post-operative physiotherapy, and consultant follow-up outpatient clinic, among others. This highlights the importance of an effective DDH screening program to improve the rate of early detection of DDH as it benefits both the patient in avoiding complex surgical intervention associated with late detection as well as the healthcare system in mitigating costs.

**Figure 4:**
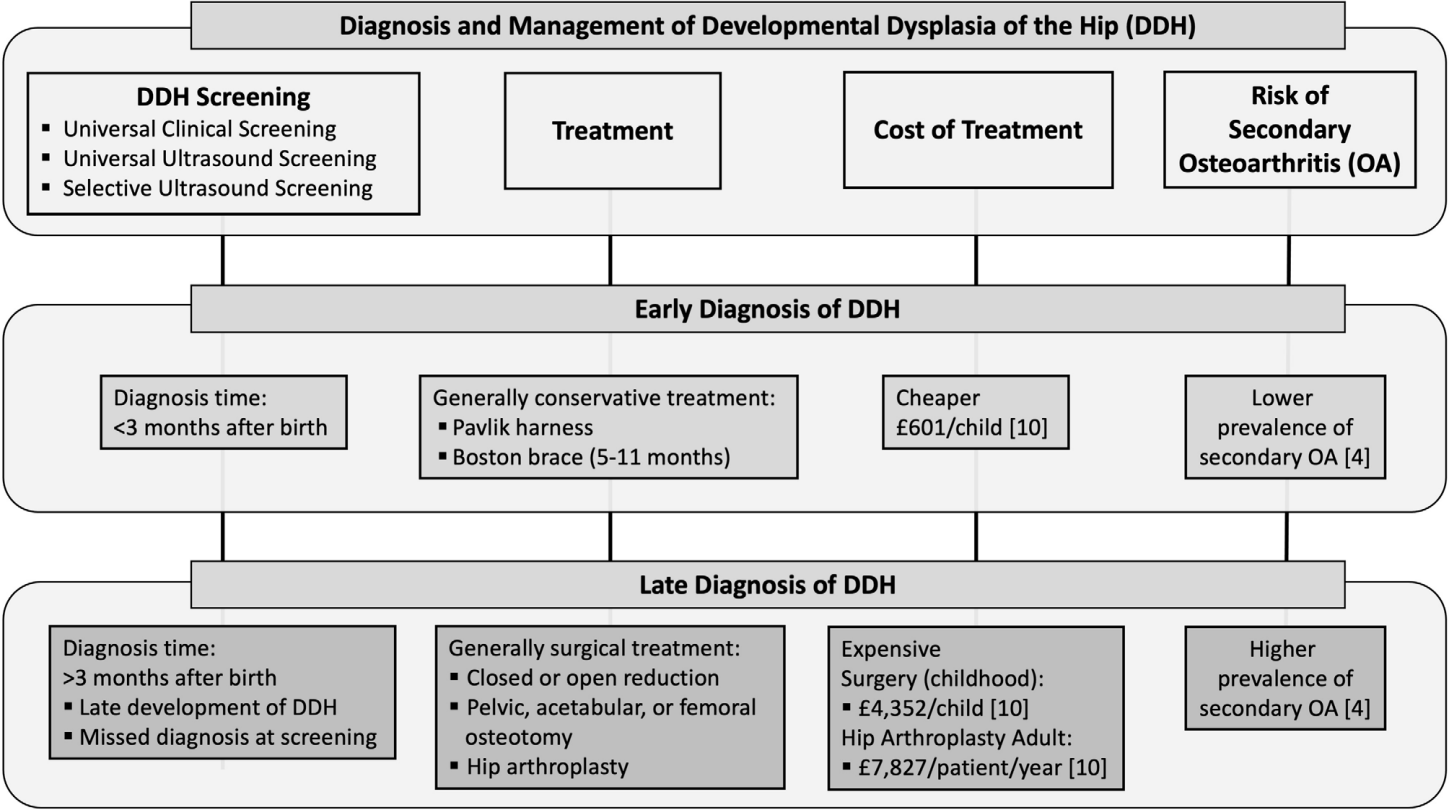
Summary of the diagnosis and management of developmental dysplasia of the hip (DDH).

An important tool to gain insight into the prevalence and treatment of late DDH is patient joint registries. Patient registries are important for research, quality improvement, and evidence-based medical decision-making [20]. Specifically, the national arthroplasty registers around the world capture data on patient demographics, procedures, devices used, and patient outcomes. The National Joint Replacement Registry of England, Wales and Northern Ireland (NJR) is the largest arthroplasty registry in the world, which makes it a valuable database to assess the demographics and clinical diagnosis of patients undergoing arthroplasty procedures. According to the NJR annual reports, osteoarthritis was recorded as the main indication for surgery in 90% of hip replacement patients in 2016 [21] and 88.8% of patients in 2017 [22]. Whether this osteoarthritis is primary or secondary due to an underlying condition is not likely recorded reliably in the registry as the surgeon or nominated deputy records the diagnosis after the procedure. It is likely that more subtle dysplasia would not be noted and would be recorded as primary osteoarthritis. DDH in adulthood causes secondary osteoarthritis and accurate classification and input of this information into the patient registry will benefit our knowledge on the prevalence of DDH and other hip pathologies causing secondary osteoarthritis in adulthood. According to the 2018 National Joint Registry (NJR) report, in 2017, osteoarthritis was an indication for surgery in 911,854 patients (91.9% of the cohort) [22]. Based on the NHS healthcare costs for 2016/2017, the annual cost per individual including the inpatient, outpatient and primary care are as follows: the year before hip replacement – £2522, the year with the hip replacement – £9295, and the year after hip replacement – £2692 [23] (Figure 4). Although the number of dysplasia patients is unknown for this dataset, it is clear that the costs for treating osteoarthritis secondary to dysplasia during adulthood with a hip replacement surgery would be much more substantial than identifying and treating these individuals in early life.

There are several limitations to this study. First, since this is a radiographic analysis, we do not know if these patients were diagnosed with DDH and received any conservative or surgical treatment prior to THA. Performing a retrospective chart review would aid in obtaining this information and establishing any cause of secondary osteoarthritis. In addition, radiographic measurements were made on the non-operated contralateral hip, and this assumes that the patients categorized as dysplastic have bilateral hip dysplasia. On the contrary, for patients who had a normal contralateral (non-operated hip), this assumes that the replaced hip was also normal and not dysplastic.

## Conclusion

Overall, despite the universal clinical screening programme with selective ultrasound screening, the prevalence of DDH is over 40% in the adult population examined in this study, as determined by radiographic analysis of patients below 50 years of age undergoing THA. Classification of primary and secondary osteoarthritis in the joint registries will benefit our knowledge of the prevalence of DDH in the adult population. Ultimately, early detection and treatment of DDH will reduce the onset of secondary osteoarthritis, thereby decreasing the need for expensive THA techniques and financially benefiting the healthcare system.

## Notes

Muddaluru V, Boughton O, Donnelly T, O’Byrne J, Cashman J & Green C (2023) Developmental dysplasia of the hip is common in patients undergoing total hip arthroplasty under 50 years of age. SICOT-J **9**, 25.
